# IMPlementation of An online Relatives’ Toolkit for psychosis or bipolar (IMPART study): iterative multiple case study to identify key factors impacting on staff uptake and use

**DOI:** 10.1186/s12913-020-5002-4

**Published:** 2020-03-17

**Authors:** Fiona Lobban, Duncan Appelbe, Victoria Appleton, Julie Billsborough, Naomi Ruth Fisher, Sheena Foster, Bethany Gill, David Glentworth, Chris Harrop, Sonia Johnson, Steven H. Jones, Tibor Zoltan Kovacs, Elizabeth Lewis, Barbara Mezes, Charlotte Morton, Elizabeth Murray, Puffin O’Hanlon, Vanessa Pinfold, Jo Rycroft-Malone, Ronald Siddle, Jo Smith, Chris J. Sutton, Pietro Viglienghi, Andrew Walker

**Affiliations:** 1 Division of Health Research, Lancaster University, Bailrigg Campus, Lancaster, LA1 4YW UK; 2 Clinical Trials Research Centre, University of Liverpool, Liverpool, UK; 3 McPin Foundation, London, UK; 4 participating NHS trust, England; 5 Division of Psychiatry, University College London, London, UK; 6 Research Department of Primary Care and Population Health, University College London, London, UK; 7 Faculty of Health & Medicine, Lancaster University, Lancaster, England; 8 School of Allied Health and Community, University of Worcester, Worcester, UK; 9 Centre for Biostatistics, University of Manchester, Manchester, England

**Keywords:** Psychotic disorders, Caregivers, Internet, Implementation science, Digital health intervention, Mental health, Case series, Early intervention

## Abstract

**Background:**

Despite the potential of digital health interventions to improve the delivery of psychoeducation to people with mental health problems and their relatives, and substantial investment in their development, there is little evidence of successful implementation into clinical practice. We report the first implementation study of a digital health intervention: Relatives Education And Coping Toolkit (REACT), into routine mental healthcare. Our main aim was to identify critical factors affecting staff uptake and use of this online self-management tool for relatives of people with psychosis or bipolar.

**Methods:**

A mixed-methods, theory-driven (Normalisation Process Theory), iterative multiple case study approach using qualitative analysis of interviews with staff and quantitative reporting of uptake. Carer researchers were part of the research team.

**Results:**

In all, 281 staff and 159 relatives from Early Intervention teams across six catchment areas (cases) in England registered on REACT; 129 staff took part in qualitative interviews. Staff were positive about REACT helping services improve support and meet clinical targets. Implementation was hindered by: high staff caseloads and difficulties prioritising carers; perception of REACT implementation as research; technical difficulties using REACT; poor interoperability with trust computer systems and care pathways; lack of access to mobile technology and training; restricted forum populations; staff fears of risk, online trolling, and replacement by technology; and uncertainty around REACT’s long-term availability.

**Conclusions:**

Digital health interventions, such as REACT, should be iteratively developed, evaluated, adapted and implemented, in partnership with the services they aim to support, and as part of a long term national strategy to co-develop integrated technology-enabled mental healthcare. Implementation strategies must instil a sense of ownership for staff and ensure they have adequate IT training, appropriate governance protocols for online working, and adequate mobile technologies. Wider contextual factors including adequate funding for mental health services and prioritisation of carer support, also need to be addressed for successful implementation of carer focussed digital interventions.

**Trial registration:**

Study registration: ISCTRN 16267685.

## Contributions to the literature

- This is the first study to examine factors affecting staff uptake and use of a digital health intervention in UK mental health services.
- Mixed methods were used to understand staff engagement with the Relatives Education And Coping Toolkit (REACT) in Early Intervention teams across six locality based services, purposively sampled for geographical and ethnic diversity in order to maximise generalisability of findings.
- Findings highlight the need to embed technology development within clinical services; embrace an iterative long-term model of development, testing and adaptation; and provide adequate mobile technology, IT training and digital governance infrastructures to support new ways of working.

## Background

Digital Health Interventions (DHIs) are increasingly being developed for people with severe mental health problems including psychosis and bipolar, to improve symptom monitoring [1], medication management [2], and access to information and support [3]. Whilst the evidence base for their effectiveness is less advanced than for other mental health disorders such as depression and anxiety, emerging data suggests that they can be feasible, acceptable, and usable [4–6], with preliminary evidence suggesting they can be as effective as more traditional, non–technological self-help interventions [7, 8].

However, evidence for successful implementation of DHIs in routine healthcare services is far more limited. Despite substantial investment, many DHIs are either not adopted by their intended users, are abandoned, fail to scale up locally or spread to other settings, or are not sustained over time [9, 10]. We urgently need to understand the key factors impacting on implementation of DHIs to improve their design, evaluation, commissioning and delivery.

A recent systematic review of 26 studies reporting factors impacting on delivery of DHIs for people with psychosis or bipolar identified the following determinants of uptake: staff and service user attitudes; complexity of the user interface; staff / peer support to use the intervention; fit with existing service IT infrastructures; and costs to development and delivery [11]. The majority of the studies reviewed were American, with only 2 in the UK, both of which were feasibility studies in a research rather than clinical context [12, 13]. Given the importance of context for implementation [14] the generalisability of these findings to routine mental health services is limited. The review found no studies of DHIs to support relatives of people with psychosis or bipolar.

Relatives of people with psychosis or bipolar provide a large amount of unpaid care [15] but at high personal cost (distress and burden) [16–18]. The UK National Institute for Health and Care Excellence (NICE) recommends that all relatives be given carer-focused education and support, and offered structured family intervention to enhance family coping and communication [19, 20]. However, audit data show services currently fall well below this target, with only 50% of relatives receiving a carer-focused education and support programme, and 12% a structured Family Intervention [21].

DHIs offer the potential for widespread dissemination of high quality, standardised care, made easily available, alongside a mechanism for uniting people online to share their experiences through peer support. Although DHI development costs can be substantial, ongoing delivery has the potential to be more cost effective in the long-term than face-to-face support and more accessible to those in rural areas and developing countries [22].

The Relatives’ Education And Coping Toolkit (REACT) is a supported self-management toolkit, providing accessible evidence-based information and support for relatives of people with psychosis or bipolar. Following evidence to support feasibility, acceptability, and effectiveness in reducing relatives’ distress as a paper-based tool [23], REACT was developed into an online resource, incorporating a peer support forum, direct messaging, and extended to relatives of people with bipolar [24]. The clinical and cost effectiveness of REACT when offered directly to relatives recruited from outside clinical services is being tested [25]. In this study we examined implementation of REACT online offered to relatives supported by staff working in clinical services in England. This is the first study to examine critical factors affecting staff uptake and use of a digital health intervention in UK mental health services. A full funders report will be available for further detail [26].

## Methods

### Design

We used a theory-driven multiple case study design [27] integrating quantitative uptake and use assessments and qualitative exploration of mechanisms.

Normalisation Process Theory (NPT) was used to guide data collection, analysis and interpretation. NPT focuses on the work done by staff to understand the processes by which a complex healthcare intervention is implemented, embedded, and integrated (or not) into practice [28]. NPT has been extensively applied in eHealth settings [29–31] allowing us to compare our findings with previous studies. The protocol was published prior to the end of recruitment [24], and StaRI guidelines [32] used for reporting.

### Context

The study took place in Early Intervention (EI) teams across six National Health Service (NHS) trusts (cases) in England. Trusts provide healthcare to particular geographical areas, and within each trust there is an EI team which provides early intervention support to people with early signs of psychosis/bipolar. Cases were purposively sampled for geographical and ethnic diversity to maximise generalisability. To protect anonymity, a multi-layered taxonomy of birds and habitats was used for trusts and clinical teams (see Table 1).

**Table 1:** Description of features of IMPART cases relevant to study

Wave	Case	Description
Wave 1	Woods	• Urban area, very high rates of psychosis • High ethnic diversity • Two geographically distinct teams • Reported average caseload per staff of 28 • Very high staff turnover & absence • Low morale: half of one team left in the first 6 months of study
	Moor	• Large rural area • Population predominantly white British • Early Intervention not separate service, embedded in geographically spread community teams • Lower caseloads (approximately 15, exact figures n/a) but long travel times
Wave 2	Ocean	• Urban area • Population majority white British • Three geographically distinct teams which performed quite differently • One team had very high staff turnover and high levels of sickness absence, in this team carer support was delegated to one carer lead rather than part of all CC’s work. • Caseloads high (approx. 26)
	Seashore	• Primarily urban area • High ethnic diversity • Three teams across locality, operating quite differently and independent of each other • Low staff morale and very high turnover, in one team all CCs and team manager left over a period of three months • Trust implementing new DHI for service users at the same time as IMPART study • Caseloads described as ‘high’ but numbers not available due to period of intense change
Wave 3	Lakes	• Largely rural area • Six teams cover large geographical area, managed in pairs • First IL a senior psychiatrist who left early in project, succeeded by another psychiatrist • Led to variable engagement with IMPART study over time
	Marsh	• Mainly urban area with rural pockets • Two separately located teams covered by one IL, one RS • Very early in project, RS role given to non-clinical staff member in R&D department

*CC Care Coordinator*

*IL IMPART Lead*

*RS REACT Supporter*

*R&D Research & Development*

### Participants

Staff identified as having specific relevant roles were invited and consented to interviews and /or attending stakeholder groups by the research team. An IMPART lead for each trust facilitated implementation of REACT and assisted with the research.

### Description of the intervention

REACT was designed with extensive involvement from relatives [33, 34] and included: 12 psychoeducation modules addressing key questions identified by relatives; peer support through a moderated group forum; a confidential direct messaging service; and a resource directory (RD). All 12 modules contained: evidence-based written information; videos of clinical experts and/or content from experts by experience to illustrate key points; and self-reflection tasks to ensure content was personalised to the user. A “Meet the Team” page ensured that relatives were fully informed about who was delivering the content of the site. ‘Mytoolbox’ offered users a confidential space to save links to any information they might want to access easily later, including specific toolkit content, self-reflection tasks, and external web links. REACT Supporters were members of the EI team, identified by the IMPART Leads, responsible for moderating the forum, responding to messages from users, updating the RD if required, and guiding users to relevant parts of the toolkit. A separate instance of REACT toolkit was set up for each case. Further details and images of the intervention can be found in the protocol paper [24].

Table 2 shows the different roles allocated within each team and their levels of site access.

**Table 2:** Roles and levels of access for each type of REACT user account

Role	Description of role	Access
IMPART lead	Provide a link between the research team and clinical service. Provide access to key data sources. Create accounts for REACT Supporters and all clinicians	Full access to REACT trust website, with information regarding all signed-up clinicians, REACT supporters and relatives. Could not access forums and direct messages.
REACT supporter	Support the relative to use and get the most out of the toolkit. Moderate the REACT Group forum and respond to direct messages from relatives. Update local information on the Resource Directory	Access to all aspects of REACT toolkit including forum and direct messages, and details of relatives who have been invited.
Clinician	Invite relative to use REACT- both verbally and by sending them an email invite.	Sign up relatives only; could access toolkit modules.
Relative	End users of REACT	Access to REACT toolkit, including forum and direct messages.

### Implementation plan

Local stakeholder groups (SGs) of relatives and staff advised on planning how REACT was introduced in each local context. IMPART leads were encouraged to customise their REACT site by adding a logo, times that the forum/direct messaging services were actively moderated and by whom, emergency contacts, and photos and biographies of the trust’s REACT Supporters and IMPART leads. IMPART leads created REACT Supporter accounts for the staff moderating the forum and direct messaging, and clinician accounts for staff who could invite relatives to REACT by email.

All trusts received pilot versions of REACT including an online manual from 19 September 2016 to 21 November 2016, during which minor edits were made. Each trust had at least one face-to-face training session, after the site went live, providing an overview of the importance of carer information and support; an outline of key components and how to use REACT; and aims of the IMPART study.

Data were collected over 18 months, first in two trusts (wave 1) in which key factors affecting implementation were identified, and in a further two (wave 2) following work done by the research team and stakeholders to design a revised implementation plan. This was repeated for the remaining two trusts (wave 3), with revisions culminating in a final list of recommendations based on understanding of key factors impacting on implementation across all six cases. Order of trusts was determined by pragmatic reasons related to the time taken for trust approvals.

### Implementation outcomes

Staff engagement with the REACT toolkit was measured by the number of accounts staff created, and number of invitations sent to relatives over the data collection period. All data were recorded on the REACT website.

### Process evaluation

Factors affecting implementation outcomes were explored through individual staff interviews; observations, document analysis; researcher reflective diaries, and Stakeholder Groups (SGs). Purposive sampling of participants was used to identify: IMPART leads (ILs), REACT supporters (RSs), team managers (TMs), and frontline clinical staff. The interview guide [35] was designed to identify factors affecting implementation of REACT. Following written consent, interviews were conducted face to face or by phone, and audio-recorded. Spradley’s [36] nine dimensions of observations was used to develop an observation proforma [35], adapted to test propositions in each context. Trust and national policy documents were sampled to help inform an understanding of case context, and whether REACT fitted into existing pathways and strategies. Reflective diaries [37] were kept by the lead researchers throughout the study and used to develop the researchers’ interpretation of the data as it was collected. SGs took place in each trust prior to the revised implementation plan being delivered, providing useful information about the implementation context, and co-developing subsequent versions of the implementation plan. Attendees in each site included IMPART Leads, service managers, frontline clinical staff, and relatives.

### Analysis

Descriptive summaries of implementation outcomes were calculated using quantitative measures of staff activity. Qualitative data sets collected from each case were analysed using framework analysis [38] supported by NVivo software [39]. Our data were first analysed within each case before we examined similarities and differences between trusts. Data analyses were undertaken in parallel with data collection to encourage iterative testing of emerging themes and inform subsequent implementation activities.

All data were read and re-read by the Research Associates (VA, BG, PO, CM, EL), and synthesised using the four main constructs of NPT [31] with guidance from senior members of the team (FL, NRF, EM). Coding frameworks, and illustrative sections of transcripts were discussed in multi-disciplinary data clinics which included all authors. Here we report the key factors identified as impacting on implementation of REACT, in relation to the following 4 NPT core constructs: coherence; cognitive participation; collective action; and reflexive monitoring.

## Results

### Evolving implementation plan

In wave 2 the implementation plan included the addition of: REACT booklets and business cards with details of how to access REACT for staff to give to relatives in face-to-face meetings; Staff office reminders with the REACT logo on including mugs and pens; automated email nudges for staff and relatives; a service planner to facilitate teams to allocate staff to key roles supporting REACT; and an auditing dashboard to show staff which relatives had been invited to use REACT. In wave 3, the plan evolved further to include a “Request Access Button” for relatives who wanted to self-refer; staff induction packs for each role; a new “REACT Champion” role to promote REACT; a revised online “how to” manual for staff including a quiz to facilitate engagement; and printable PDF versions of REACT modules to offer as “tasters” for the website.

### Implementation outcomes

Table 3 provides summary statistics for implementation outcomes in each case.

**Table 3:** Implementation outcomes over 18 months

Wave	Wave 1		Wave 2		Wave 3		
Trust	Woods	Moor	Ocean	Seashore	Lakes	Marsh	Total
No. of clinician accounts created	44	37	32	63	64	41	281
No. of clinicians sending invites (% of clinicians who created account)	8 (18)	12 (54)	4 (12)	18 (29)	8 (13)	7(17)	57
Median (range) invites sent per clinician	3 (1,11)	3 (1,9)	8 (4,20)	2.5 (1,25)	2 (1,15)	3 (1,45)	3 (1,45)
Total no. of Invites sent	35	47	40	112	29	92	355
No. of relatives invited (% of caseload)	29 (6)^a^	40 (18)^a^	37 (5)^a^	93 (24)^a^	25 (4)^b^	86 (23) ^c^	310
No. of relatives accounts created (% of caseload)	7 (1)^a^	24 (11)^a^	20 (3)^a^	38 (9)^a^	17 (3)^b^	53 (15)^c^	159
No. of staff interviewed	15	15	42	23	20	14	129

a Source for caseload is trust self-assessment for CCQI National Early Intervention in Psychosis Audit 2016–17

b Source for caseload is EI Access and NICE Concordance Presentation by EI Clinical Lead

c Source for caseload is EI Provider & Commissioners’ Report 2016

In all, 281 staff registered on REACT and between them, sent 355 invitations to 310 relatives, and 159 relatives registered. The highest number of clinician accounts were created at Lakes Trust (wave 3), least at Ocean (wave 2). Seashore Trust (wave 2) sent the most invitations to relatives, while Lakes Trust sent fewest. A minority of clinicians across all trusts sent invites, with the fewest being in Ocean, and most in Seashore. There was no evidence of an increase in implementation outcomes across the 3 waves, or as a result of the evolving implementation plan, though the timeframe of this analysis may have been too limited to adequately assess this. The aim of this study was to understand the factors determining these numbers, rather than to maximise them.

### Process evaluation – key factors impacting on implementation

Table 4 summarises the key factors impacting on implementation, with illustrative quotes from across all six cases.

**Table 4:** Key factors impacting on implementation of REACT

NPT components	Key factors impacting on implementation	Illustrative quotes
Coherence	Generally good understanding of REACT. Staff could identify benefits to relatives and staff including: a) standardised information,	**Woods, Clinician:** One potential benefit is you can very easily offer I suppose everyone with an internet connection really the same information…consistently across the board…And hopefully if it’s used then you can get used to working with a family that have a certain knowledge base which I think would be good.
	b) available any time, any place, anywhere;	**Woods, IMPART Lead:** [Groups] can be very responsive and warm and everything but they’re also yeah you have to get here for 6 o’clock on alternate Tuesdays… [A website is] much more kind of accessible and people can read stuff in their own speed.
	c) training for staff;	**Seashore, Clinician**: …it’s nice to know oh right this is something I have that’s up my sleeve and it kind of gives me a sense of like a purpose […] now I feel like yeah I’m a specialist in carers’ needs and I have a specialist tool for you and I think it’s really useful.
	d) hitting national targets;	**Woods Clinician:** Yeah I think it fits in in terms of reaching the family interventions target and as I said before I think it fits in with the BFT [behavioural family therapy] as well. So it kind of hits a couple of the targets that we’ve got to do I think.
	e) reducing staff workloads;	**Marsh, Clinician**: Yeah I think it’s really good, it’s brilliant, especially as with all the will in the world you’ve only got a certain amount of time when you can do stuff with carers and you’ve got everything else, in that way it’s a really educational tool that anyone can use you know, it’s really good.
	f) empowering self- management for relatives	**Lakes, REACT Champion**: I mean relatives would like to look at it so they can support their own child or son, daughter or whoever you know, their husband, wife, they might need those services and they can take a lead role it in.
	g) good fit with other parts of carer services	**Ocean, Clinician**: Yeah, for instance I’m working with a family that’s a bit there’s quite a lot of conflict and it’s a bit split up at the moment, so my client’s a male client, he was living at home, his mental health brought then such a degree that it was impacting on his marriage and his relationship with his children, so he had a bit of respite over with his mum, so I kind of offered them BFT family intervention with the children and the wife and the grandma, and also offered them access to the REACT website for them to access more information around psychosis and their understanding of the difficulties that the son and husband were going through, to kind of get an understanding about some of the complexities and difficulties and about treatment.
	**BUT**	
	REACT seen as research, rather than part of the clinical service. This generated some short-term behaviour but impeded long-term commitment as seen as inherently time-limited and the responsibility of the R&D team.	**Seashore, IMPART Lead**: …I think if REACT had been introduced as not research but just something we’re going to do from now, that by the way we might evaluate, possibly it might have been seen as more of an infrastructural thing, this is what we will do, but yeah. The kiss of death is kind of true, but people switch off as soon as you go this is a person from X university and they’re going to talk about this project. You can see half the team sort of go oh God and fold their arms and how many more minutes have we got to put up with this, no matter what the project is…
	Challenges with delivering face to face training to all staff due to shifts, staff turnover etc. Online training manual not well used. Consequently, some staff not familiar with REACT and didn’t understand their role in offering it.	**Lakes, Clinician:** … I can’t recall anyone going through the modules clearly with us, I think it was like your team signed you up like so [IMPART Lead] as the lead just sent everyone log ins and said off you go, have a look at it yeah, hence why I was thinking some team time to look at it together, so we all feel confident, we can ask questions.
Cognitive participation	“Professional ownership” was important in determining who used REACT	**Lakes, Clinician:** Yeah I do think there is something a bit odd about a family resource coming from psychiatry not from the family therapists; I do think that is unusual.
	Staff feared negative impact on relationships with relatives in case digital support would not be as effective as face-to-face support and relatives may feel “fobbed off”;	**Woods, IMPART Lead**:…there’s this feeling I think both among the managers and the staff, they don’t quite seem to see a digital intervention as something that’s sort of real and high quality, I think they kind of feel that they’re offering relatives something that’s a little bit sort of weird and tricksy and not quite the real stuff that they as mental health professionals are supposed to be offering and I think that’s quite sort of wide spread, almost just like embarrassment in offering this.
	REACT may not be appropriate for everyone – particularly older adults	**Moor, Clinician**: ..I don’t have a lot of the younger people on my case load, I have the older end, in that you know some of them are in their late 50s coming up to some of them even just going up to 60 age bracket, so even though I try to take my information leaflet along with me and say what REACT is about, and a very, they seem to have accepted that it’s a useful resource, very you know sort of very kind of me to offer it, but because they’re not clued up with computers, that computer age and literacy, they decline, they say thank you but no thank you, we don’t want to bother with it.
	REACT content static and sometimes at odds with current practice e.g. REACT provides definitions for the diagnostic terms that relatives may have come across (based on what relatives identified as useful things to include).	**Marsh REACT Champion:** I think the first description was one of schizophrenia and I wasn’t impressed with that ‘cos I think that is scary for having that as your first introduction to psychosis…We wouldn’t ever give somebody a diagnosis of schizophrenia within the first year probably anyway.
	REACT roles allocated, but no choice and not volunteered.	**Lakes, Clinician:** What I’ve done in Grebe is I have a carers’ champion, and then I have [name] who’s the React supporter, and I’ve actually paired them up so that they support each other, because there’s a lot of work to be done through the carers’ champion that could be useful to React, so it was easier to do that, so they’re both doing a dual role, so I think that might be something that I’ll do in the team, but again we’re both small teams and it can be a lot of work you know
	Negative experiences of IT systems in all trusts, leading to negative attitudes towards IT in general.	**Woods, IMPART Lead**: I think also all the IT systems at work are pretty awful really, we’ve got terribly slow internet and frequent problems logging in to the most basic things, and I think there’s something about the sort of rubbish nature of the IT systems and you’ve seen the phones the staff get given that don’t really do the internet, and I think there’s something about all that that sort of I think it just creates a mind-set where you know people may be sort of inhabitants of the digital world outside work but somehow at work it doesn’t really seem like we are in that age…
Collective Action	Despite recognising value, REACT was difficult to prioritise. In the context of limited resources, priority was given to activity	
	a) focussed on service user outcome,	**Ocean, Clinician**: I have given it to a couple of people when I’ve done first assessments and yeah it probably isn’t seen as a priority with me…You go in and start work and developing care plans and things with the clients [and] maybe you don’t always then focus on the carer.
	b) linked to financial incentives	**Lakes, REACT Champion:** If we’ve got our managers saying well…there’s £20 m to this physical health target…it’s quite disheartening for the nurses because all you want to do is serve your patient and in essence you’ve got to come off clinical duty so you can prioritise this target…There’s money attached to absolutely everything.
	Changing and competing externally driven priorities made strategic planning for new interventions difficult	**Woods, Clinician**: I think there’s an awful lot of new stuff happening right now, which makes it quite difficult for people to know what they should be focusing on […] you know one week we’re saying this is the absolute priority, and then the next week we’re saying, actually that’s no longer absolute priority.
	Most teams had high caseloads, low staffing levels and poor morale.	**Seashore, IMPART Lead**: The manager in my team is very supportive of it, but I think it’s care coordinators being over-worked by other demands you know. We’ve got someone leaving now and we’re not allowed to have a locum in place of them, they’ve got more caseload, they’ve got to do the CPAs, they’ve got to do the safeguarding, they’ve got to do the…. Well those are subjects, that feels more a priority than signing people up…
	REACT not sufficiently user friendly or robust for staff	**Seashore, Clinician:** I think everyone would be able to use it, would have the kind of capability to use it, it would just be getting that initial like getting on it and having I think the log ins and things like that were a little bit difficult because everyone’s got so many log ins for different things, and people forget their passwords and that makes it a little bit more difficult.
	REACT did not always work on the IT equipment available to staff	**Moor, REACT Supporter:** …..there was one bit I couldn’t get on because of the browsers that we use, but that’s an issue. …….[..]..I’d have to get on and show you, but that’s an issue internally…It’s unsupported, I need a better browser. I get that with anything external and I have got Chrome, so I don’t know whether that would.
	Online was “out of sight, out of mind” for staff	**Woods IMPART Lead:** People just forget it…When you’re doing an assessment, when you’re meeting a family member, you have a thousand and one things on your mind that you’d like to assess and write down and stuff and it’s difficult to remember the spiel.
	React Supporters not confident or supported to respond to forum or direct messages online….,	**Marsh, Clinician**: I think that’s probably still a grey area because so far I think what our trust is good at is managing sort of clinical risk […] Online is a different thing, I think we’re not exposed to it enough and therefore we haven’t outlined how that would work, so I think you know managing a forum, as far as I’m aware there’s no formal training for that in the trust, so that’s quite bespoke.
Reflexive monitoring	Feedback from relatives was very important in motivating staff behaviour.	**Ocean REACT Supporter:** I’ve had such nice feedback from people really, relatives really sort of saying I’m so glad you told me about this, ‘cos I’ve been Googling and trying to find stuff out and it’s all just coming back and I don’t understand it, whereas this is kind of it’s easier to access and it’s user-friendly I think.
	Lack of feedback from relatives and low activity on forum were demotivating for staff.	**Marsh Clinician**: So we did discuss it a while ago and I remember people were saying that they weren’t sure whether the people were actually using it or not, you know whether we can see that or not, which would be good to know, if people are using it.

REACT had reasonable coherence for staff, in that they clearly understood what the toolkit was for, who it was aimed at, and the potential benefits for both staff (skills development, meeting national targets [40], facilitating other family interventions) and relatives (access to standardised information at convenient time and location that empowers them to self-manage). Staff could see how REACT complemented other interventions, such as Behavioural Family Therapy (BFT), offered by the team. However, a key problem was that implementation of REACT was generally seen as research, rather than routine care and consequently may have been perceived as time-limited, and the responsibility of the research team.

“*…I think if REACT had been introduced as not research but just something we’re going to do from now, that by the way we might evaluate, possibly it might have been seen as more of an infrastructural thing, this is what we will do, but yeah. The kiss of death is kind of true, but people switch off as soon as you go this is a person from X university and they’re going to talk about this project. …” (Seashore, IMPART Lead)*

An additional coherence problem was due to high staff turnover, disparately located teams, and preference for face-to-face training. Consequently, while staff understood the general purpose of REACT, many were not familiar with the detailed content and lacked confidence in promoting it to relatives.

“*I deliver it to all my patients’ families. But I’m not really sure how to deliver it still. …….What it is, you know what do we call it, what is it, is it information kind of site, or I don’t really know how to deliver it…………Possibly, yeah I haven’t had the training and no one’s really told me what it exactly is.” (Moor, Clinician).*

Mitigating strategies included delegating the role of inviting relatives to a small number of individuals (as in Marsh), and appointing a REACT champion to remind clinicians about REACT (Seashore, Marsh & Lakes).

Coherence problems tracked through to cognitive participation. Staff agreed REACT could be of value to relatives, but only if delivered as part of a comprehensive care package, including face-to-face support. Opinions differed over who should have ownership over the delivery of REACT, depending on how the services were structured, and how REACT had been introduced. This impacted on buy-in from individual staff.

*“It shouldn’t be psychology should it, REACT, it’s just an information sharing but because it is held in psychology people are thinking oh it’s another psychology strategy.” (Seashore, Clinician).*

REACT was designed as an adjunct to a comprehensive service. However in trusts struggling to deliver services for relatives, staff feared relatives might become frustrated when they learnt from REACT about services they should have access to, but did not. Across professions, there were concerns about digital interventions replacing face-to-face therapies for psychosis, and particularly for older adults, who staff perceived as less likely to engage with DHIs.

*“I suppose it is a slight break with tradition isn’t it, to use an internet-based source, because I suppose it depends how professionals are; our most valuable tool is ourselves and I don’t know whether there’s something that potentially is a bit daunting about handing that over to something else, i.e. technology” (Marsh, Clinician).*

Commitment within clinical teams to integrate REACT into clinical practice was limited. REACT was rarely a regular item on any clinical meeting agendas. Tasks were usually allocated, not volunteered for, and training and supervision of key roles including the REACT Supporter role was lacking.

For all trusts, the major challenges with implementation were located in the area of collective action, and how the introduction of REACT was managed through allocation of resources, and execution of protocols, policies, and procedures. Staff in all trusts were under great pressure, with high caseloads, competing priorities and, in some trusts (Woods and Seashore) high staff absence and turnover. All staff were working at (or beyond) capacity, lacking resources to deliver REACT. Staff felt forced to prioritise within priorities, leading to concentration of efforts on measureable outcomes (service user contacts rather than carer contacts), with financial incentives attached. As the Moor Trust IMPART lead put it during one of the SG meetings, “*REACT is just not the shark nearest the boat*”.

*“I think there’s an awful lot of new stuff happening right now, which makes it quite difficult for people to know what they should be focusing on […] you know one week we’re saying this is the absolute priority, and then the next week we’re saying, actually that’s no longer absolute priority” (Woods, Clinician).*

It was unclear to researchers or clinical staff who was responsible within trusts for the strategic direction of service development and who could facilitate the shift in priorities needed for staff to embrace novel interventions such as REACT.

A further barrier to action was disjunction between the online nature of REACT (“out of sight and out of mind”), and staffs’ primarily paper-based and community-located ways of working. This was exacerbated by lack of up-to-date mobile technology, and previous negative online experiences including a staff member having been “trolled” (individually named and publically criticised) on a different online forum.

Staff who did try to use REACT, reported good interactional workability, promoting better consultations with relatives. However, some content was felt inconsistent with other aspects of the service (particularly the use of diagnostic terms), and the REACT dashboard used to create relatives’ accounts was not sufficiently user friendly. The need for staff to sign into REACT using a unique username and password also caused difficulties, as staff were required to remember multiple versions of these for different online systems across the NHS. Many staff did not have access to health-service mobile technology and could not show relatives the REACT website during home-based consultations.

*“And then we can go out then, I use my own personal phone ‘cos obviously my work phone doesn’t let me go on the internet, or I’ll ask them to get their iPad out and I’ll show them that way.”(Ocean, Clinician).*

Although most relatives had personal computers or tablets, staff did not feel it was appropriate to ask relatives to log in to their own desktop computers to be shown the site, particularly as these were often in the bedroom.

Finally, staff allocated to REACT Supporter roles did not feel confident or supported to moderate the online forum or respond to direct messages. Lack of clarity over who had clinical responsibility for REACT and absence of trust policies about managing risk online, resulted in poor relational integration (understanding of accountability and confidence in each other in delivering the practices required) and consequently lack of proactive engagement by REACT Supporters. Fear of being held responsible for risk was widespread across all trusts and in one (Lakes), led to some staff withdrawing support for REACT. No trusts seemed able to reassure staff or produce risk policies adapted for online communication.

*“……it’s absolutely no chance I’m going to be putting my registration at risk to look over something that’s,…. but for the confidence, the security, the even if everybody.. somebody just says I need help right now doesn’t get it within 20 minutes, a stereotype saying [?] off the bridge and then well they put a cry for help knowing REACT Champion was on duty and he didn’t see it.” (Lakes, REACT Champion).*

Where staff overcame these barriers and invited relatives, they were frustrated by the lack of feedback (reflexive monitoring) available from relatives about the site. Where staff sought feedback directly from relatives, this was generally positive about the module content, particularly hearing the stories of others. However, relatives were disappointed by the lack of forum activity. This was partly due the small population of relatives using the site, so no-one wanted to be the first; and partly due to lack of staff promotion of forum activity. As staff became aware of the lack of forum activity, they became less motivated to invite relatives, creating a vicious cycle.

*“I think it’s just not seeing any kind of outcomes coming from registering people and it looks like there’s no activity on the site, I guess I mean people might be logging on, the few that have actually registered, but I guess there’s no way of monitoring it, no way of knowing that people are actually finding it helpful or useful” (Woods, REACT Supporter)*

## Discussion

This is the first study that examined in detail the process of implementation of a DHI aimed at supporting relatives of people with psychosis or bipolar within national publicly funded mental health services. Staff engagement with REACT was facilitated by a good fit between the rationale and design of the toolkit, and the need for them to deliver support to carers as part of audited national clinical guidelines. The toolkit was easy to integrate alongside other services currently being offered, and staff could easily see the benefits of this approach in terms of accessibility to high quality information and support for relatives and staff. Positive feedback from relatives was a strong motivator of engagement. Barriers to implementation included high staff caseloads and difficulties prioritising supporting relatives; technical difficulties using REACT; poor interoperability with trust IT systems and care pathways; lack of access to mobile technology and IT training; restricted forum populations leading to low levels of use; staff fears of managing risk, online trolling, or replacement by technology; and uncertainty around REACT’s long-term availability.

Some of these factors are consistent with the findings of previous research into implementation of DHIs into physical healthcare settings [29, 41–43] and more recently in a review of studies in mental health settings [11]. We additionally identified three key factors in relation to DHIs in mental health care settings, with clear clinical implications.

The first is the significant impact of wider social context around mental health. Despite government initiatives to achieve parity of esteem for mental and physical health [44], mental health services in this study were chronically underfunded, staff morale was low, and there was high staff turnover and absences. Staff were managing many competing priorities, and supporting relatives was not activity that was systematically recorded. Successful integration of DHIs into clinical practice requires significant engagement of time and effort from staff across all levels of the organisation. This demands an adequately funded service with the capacity to flex sufficiently to accommodate changes in practice.

The second is that digital confidence, competence, governance, and access to equipment were limited, suggesting that implementation of any new DHI would have been challenging. Managing risk was a high clinical priority, but risk management policies did not include online activity, leaving staff unclear of their responsibilities. Investment is needed to ensure staff have access to the mobile hardware they need, integrated IT platforms that support single login, and digital skills training. Governance issues around digital risk and responsibility urgently need to be addressed with clear staff policies.

A third key barrier was staff perception that REACT was a research study, rather than a clinical initiative, despite extensive attempts to explain that the implementation focus of the work. This perception was compounded by the fact that the decision to adopt REACT was often made first by research leads not clinical teams. This impeded staff uptake, and was further compounded by lack of long-term funding for REACT, meaning there was no guarantee it would be available after the study. DHIs, such as REACT, need to be co-developed and iteratively evaluated, adapted and refined with extensive staff and service user input, as part of a long-term and resourced strategy to develop fully integrated technology-enabled services [45]. The current model of develop, test, implement, can lead to promising technologies being abandoned early, as staff will quickly disengage from externally delivered technology that does not immediately work for them. Integrated and embedded technology development in clinical services will also ensure that investment in DHI development is targeted at clinical need, driven by “pull” from staff and service users, rather than “push” from technology developers.

Based on this study, we have outlined generalizable recommendations for successful implementation of DHIs, using REACT as an example (see Table 5).

**Table 5:** Recommendations for implementing digital health interventions

Understanding context is key	
Clarify exactly how DHI fits into the broader clinical service including care pathways and auditing targets	
Understand, acknowledge, and where possible address important wider contextual barriers to uptake and use of DHI	
Clarify the organisational structure and identify relevant decision-makers across different levels of the organisation, and understand how decision making around adoption of new practices happens within each organisation	
Consider which elements of DHI require local adaptation (e.g. resource directories of other available services), and which require national integration (e.g. online forums)	
Design DHI to be compatible with the range of hardware and software currently used across the healthcare teams	
Identify other organisational changes that are occurring simultaneously and consider how these may affect implementation of DHI. Simultaneous implementation of other DHIs may be particularly challenging for staff	
Maximise initial buy-in and continued use	
Clearly identify originators/developers of DHI, and seek endorsement from credible parent organisations, and end users	
Ensure DHI is clearly identified as a clinical initiative and not identified as primarily research	
Explicitly identify and label DHI with the key value(s) for each stakeholder group in the organisation	
All relevant user groups (including staff) should have full access to the DHI, pilot the DHI, consider the pros and cons, and be part of the service decision to adopt	
All staff fears and concerns about DHI should be identified and addressed in organisational written policy and staff training prior to adoption	
Promote DHI as part of a multichannel service to avoid “out of sight out of mind”	
Ensure ongoing training and support for all staff	
Consider appointing one or more champions to coordinate organisational activity and be a point of contact for DHI providers	
Train staff in all relevant skills including generic IT skills, using multi-channel, flexible training that can accommodate constant turnover of staff.	
Provide relevant feedback and manage expectations	
Audit reports around DHI use need to be easily available to stakeholders in user friendly form, with clear mechanism established for addressing feedback involving DHI providers	
Specific short and long term targets should be set regarding uptake and use of DHI to manage staff expectations and evaluate progress	

### Study strengths and limitations

The design was collaborative and informed by perspectives of all relevant stakeholders. Relatives and clinical staff formed part of our study team and contributed to design, data collection, and analysis. Stakeholder Groups were set up at each NHS trust, and played a key role in providing data and creating recommendations. Generalisability of findings was enhanced through use of multiple data sources, collected across multiple real world cases, sampled for diversity, and data analysis was strongly embedded in implementation theory. NPT proved a useful framework to identify important factors impacting on the day to day work done by staff to implement REACT.

The main limitations were the relatively short timeframe (18 months) of data collection, restricting focus to the early stages of implementation and precluding an understanding of embedding, integration, or the impact of the evolving implementation plan; and the dual role of the research team in developing REACT whilst also collecting data to understand the process of implementation. Further research needs to test the generalisability of the factors identified as impacting on implementation of REACT in mental health services, to other DHIs and within other healthcare systems, and the effectiveness of the recommendations. Further research could also explore how individual differences among staff impact on levels of engagement, which has not been addressed here. This study has focussed only on uptake and use by staff, but equally important are the factors impacting on uptake and use by relatives who were offered REACT, and these will be reported elsewhere. There are many alternative theoretical frameworks that could have been used and of particular interest is the more recently proposed non-adoption, abandonment, scale-up, spread, and sustainability (NASSS) framework which has been specifically developed for DHIs [46]. NASSS was not available at the start of this study but given the specificity of focus on implementation of DHIs, and the significant role that the wider context played implementing REACT, this may offer a useful framework for future work in this area.

## Conclusions

In the first implementation study of a DHI (in this study REACT) in the UK NHS mental health services, we identified many factors across staff coherence, cognitive participation, collective action and reflexive monitoring, that impact on the success of implementation and which are likely to be relevant to other DHIs in mental health. Some of these factors could be addressed by facilitating a model of DHI development that is strongly embedded in services, prioritises long-term intervention evolution and change, and manages staff expectations around uptake and effectiveness. However, wider contextual factors including inadequate funding for mental health services; lack of prioritisation of working with carers, and dissociation between research and clinical practice also need to be addressed.

## Data Availability

Extensive anonymised quotes from data collected during the study have been included in the paper and more detailed summaries of each case will be published as part of the study funders report. Access to further available anonymised data may be granted following review and if appropriate agreements are in place. Please contact corresponding author.

## Abbreviations

BFT: Behavioural Family Therapy
DHIs: Digital Health Interventions
EI: Early Intervention
IL: IMPART Lead
IMPART: Implementation of an online Relatives’ Toolkit
IT: Information Technology
NASSS: Nonadoption, abandonment, scale-up, spread, and sustainability
NHS: National Health Service
NICE: National Institute for Health And Care Excellence
NPT: Normalisation Process Theory
REACT: Relatives’ Education And coping Toolkit
RS: REACT Supporter
SG: Stakeholder Groups
StaRI: Standards for Reporting Implementation Studies
UK: United Kingdom
